# Dengue Transmission

**DOI:** 10.1289/ehp.0800227

**Published:** 2009-02

**Authors:** Thiruppathi Mariappan

**Affiliations:** Vector Control Research Centre, Indian Council of Medical Research, Pondicherry, India, E-mail: tmappan@gmail.com

In “Dengue Reborn: Widespread Resurgence of a Resilient Vector,” Phillips (2008) highlighted the relevant message to alert people involved in dengue and chikungunya control programs, as well as researchers. As a scientist in vector control in India and abroad for the past three decades, I would like to add valuable information.

Because the biology and behavior of *Aedes aegypti* and *Aedes albopictus* have been understood for several decades, national programs of affected countries need to adopt feasible and practical approaches to reduce indigenous transmission of dengue and chikungunya. The vectors have been well established in different geographical regions for more than two centuries because of commercial activities (Mariappan et al. 2008; Schliessmann and Calheiros 1974).

I have observed adult *Aedes* mosquitoes inside houses, in bathrooms, and kitchens; around trash; in rooms containing fiberglass water tanks; under staircases; on furniture, curtains, and hanging clothes; on ornamental and decorative objects in verandas and halls; and in garages. In Jeddah, Kingdom of Saudi Arabia, 300-year-old buildings with two- to three-floor flats and wooden-shuttered windows provide dark, cool, humid microclimatic situations ideal for *Aedes*; these buildings may house six to eight families. These occupants are often ethnic heterogenic populations including Egyptians, Filipinos, Pakistanis, Bangladeshis, Indians, Indonesians, Sri Lankans, Yemenis, and Africans.

People living in endemic belts of dengue and chikungunya frequently visit their countries and return infected with dengue, acting as potential sources for local transmission because of the high density of *Aedes* adults. Each infected *Aedes* completes its lifecycle within one apartment by taking blood from a host and depositing eggs in suitable habitats. Because of the intermittent supply of piped water (25–35 days), residents must store water in 20–40 drums holding 20–200 L each; drums are usually dark blue, black, or green to prevent growth of algae. These containers are obtained from markets or from shops that sell drums and barrels previously used for storing other materials. *Aedes* breeding has been reported in water stored in this type of recycled drums (Mariappan 2008). In addition, people from various continents who participate in the Islamic Hajj pilgrimage (visiting Mecca and Madinah) may also spend time in Jeddah, thus facilitating indigenous transmission of dengue (Fakeeh and Zaki 2003).

I fully agree with Phillips (2008) that changes in global warming lead to an increase in different vector-borne diseases. Cumulative factors are involved in the increase of dengue, including air travel, where infected individuals travel quickly from continent to continent. The risk of travelers being bitten by *Aedes* during transit is common.

Singapore, which has continued to fight against dengue for several decades, has been unable to become free from both dengue and chikungunya because of enormous natural and manmade habitats. In many cities (e.g., Rio de Janeiro, Brazil; Bangkok, Thailand; Jakarta, Indonesia; New Delhi, India), monsoons facilitate a high density of *Aedes* adults, therefore maintaining dengue transmission. In India, as many as 38 people have died from dengue, and > 2,800 have been affected (Dhar 2008).

In Jeddah, dengue cases are reported throughout the year, when there is no rainfall and during periods of occasional rain in December. Jeddah remains warm in winter, with temperatures ranging from 15°C (59°F) at midnight to 25°C (77°F) in the afternoon. Summer temperatures are considered very hot, breaking the 40°C (104°F) mark in the afternoon and dropping to 30°C (86°F) in the evening (Mariappan 2008).

Increasing awareness of improved water-storage practices among affected populations could drastically reduce the density of *Aedes* adults. Training student volunteers to control *Aedes* populations (both immature and adult) by adopting “practical hand-in-hand training” procedures in field operation has resulted in a decrease in the number of dengue cases (Mariappan 2008). Training on *Aedes* control measures using 2–3 day “train the trainers” courses has been highlighted by the European Centre for Disease Prevention and Control (Senior 2008).

Phillips (2008) illustrated the disease of poverty and reported on methods of curbing dengue’s expansion. Together, the movement of infected persons and the diurnal biting habits of vectors increase the infected vector populations, making local transmission easier. Each infected adult mosquito could infect many people in a given area before its death. In addition, eggs deposited by *Aedes* could survive > 6–8 months in adverse conditions within areas of human habitation, and those dormant eggs deposited in objects/containers may be transported to various areas through the sale and use of recycled containers used for water storage.

The increase of dengue (and dengue hemorrhagic fever) worldwide is everyone’s responsibility. Therefore, we should initiate control measures (especially in reducing sources) against vectors until a successful vaccination can be produced.
